# 1-(2,2-Di­chloro­acet­yl)-3-ethyl-2,6-di­phenyl­piperidin-4-one

**DOI:** 10.1107/S1600536813010957

**Published:** 2013-04-27

**Authors:** P. Sugumar, R. Kayalvizhi, P. Nirmala, S .Ponnuswamy, M. N. Ponnuswamy

**Affiliations:** a Centre of Advanced Study in Crystallography and Biophysics, University of Madras, Guindy Campus, Chennai 600 025, India; b Department of Chemistry, Government Arts College (Autonomous), Coimbatore 641 018, India

## Abstract

The asymmetric unit of the title compound, C_21_H_21_Cl_2_NO_2_, contains two independent mol­ecules that show similar geometrical features. The piperidine ring adopts a distorted boat conformation. The phenyl rings substituted at the 2- and 6-positions of the piperidine ring are oriented at angles of 65.4 (1) [64.7 (2)°] and 89.2 (1)° [86.3 (2)°] with respect to the least-squares plane of the piperidine ring. In the crystal, adjacent mol­ecules are linked by a network of C—H⋯O inter­actions, forming a *C*(6) chain along the *c*-axis direction.

## Related literature

For the biological activity of piperidine derivatives, see: Aridoss *et al.* (2009 ▶); Nalanishi *et al.* (1974 ▶); Michael (2001 ▶); Pinder (1992 ▶); Rubiralta *et al.* (1991 ▶). For puckering parameters, see: Cremer & Pople (1975 ▶). For asymmetry parameters, see: Nardelli (1983 ▶).
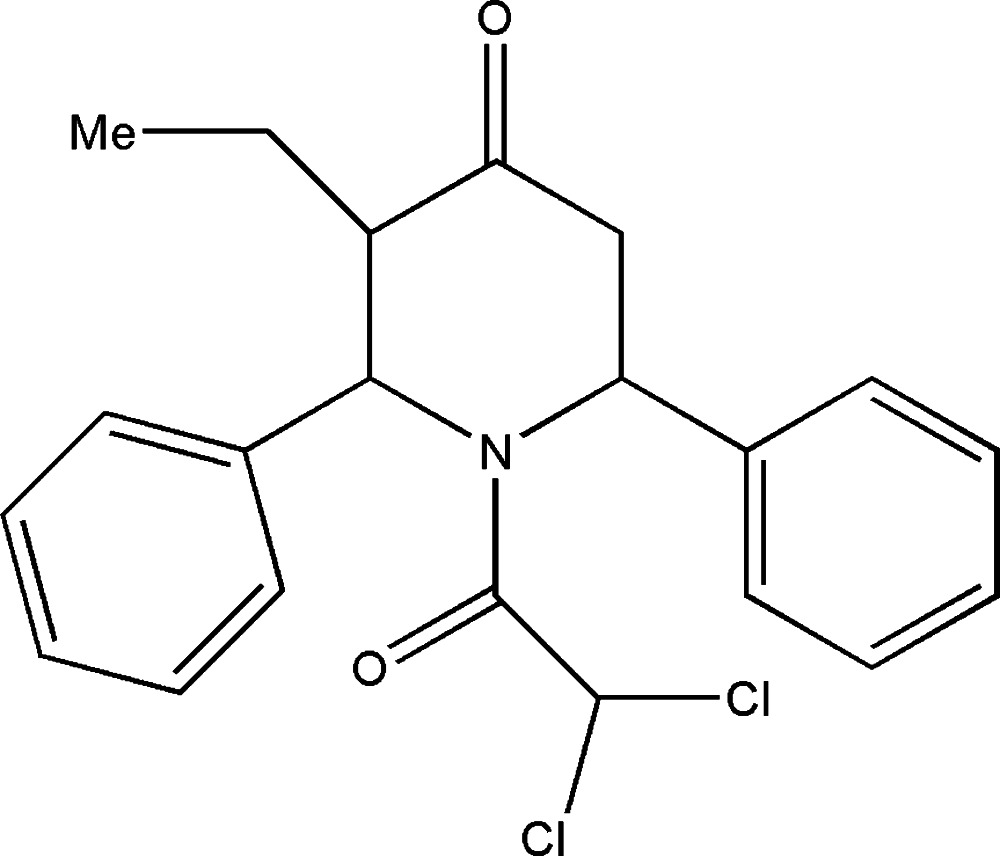


## Experimental

### 

#### Crystal data

C_21_H_21_Cl_2_NO_2_
*M* *_r_* = 390.29
Monoclinic, 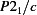

*a* = 17.4615 (13) Å
*b* = 19.0291 (15) Å
*c* = 11.9501 (9) Å
β = 91.825 (5)°
*V* = 3968.7 (5) Å^3^
*Z* = 8
Mo *K*α radiation
μ = 0.34 mm^−1^
*T* = 293 K
0.22 × 0.20 × 0.18 mm

#### Data collection

Bruker SMART APEXII CCD diffractometer
Absorption correction: multi-scan (*SADABS*; Bruker, 2008 ▶) *T*_min_ = 0.928, *T*_max_ = 0.940
36640 measured reflections
9905 independent reflections
5029 reflections with *I* > 2σ(*I*)
*R*_int_ = 0.058

#### Refinement

*R*[*F* ^2^ > 2σ(*F* ^2^)] = 0.054
*wR*(*F* ^2^) = 0.175
*S* = 1.00
9905 reflections
471 parameters
H-atom parameters constrained
Δρ_max_ = 0.40 e Å^−3^
Δρ_min_ = −0.44 e Å^−3^

### 

Data collection: *APEX2* (Bruker, 2008 ▶); cell refinement: *SAINT* (Bruker, 2008 ▶); data reduction: *SAINT*; program(s) used to solve structure: *SHELXS97* (Sheldrick, 2008 ▶); program(s) used to refine structure: *SHELXL97* (Sheldrick, 2008 ▶); molecular graphics: *ORTEP-3* for Windows (Farrugia, 2012 ▶); software used to prepare material for publication: *SHELXL97* and *PLATON* (Spek, 2009 ▶).

## Supplementary Material

Crystal structure: contains datablock(s) global, I. DOI: 10.1107/S1600536813010957/ng5321sup1.cif

Structure factors: contains datablock(s) I. DOI: 10.1107/S1600536813010957/ng5321Isup2.hkl

Supplementary material file. DOI: 10.1107/S1600536813010957/ng5321Isup3.cml

Additional supplementary materials:  crystallographic information; 3D view; checkCIF report

## Figures and Tables

**Table 1:** Hydrogen-bond geometry (Å, °)

*D*—H⋯*A*	*D*—H	H⋯*A*	*D*⋯*A*	*D*—H⋯*A*
C2—H2⋯O1^i^	0.98	2.49	3.435 (3)	161
C20′—H20*F*⋯O1^ii^	0.96	2.48	3.415 (4)	164
C6′—H6′⋯O2′^iii^	0.98	2.51	3.292 (3)	137

Symmetry codes: (i) 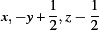; (ii) 
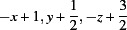
; (iii) 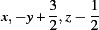.

**Fig. 1.**
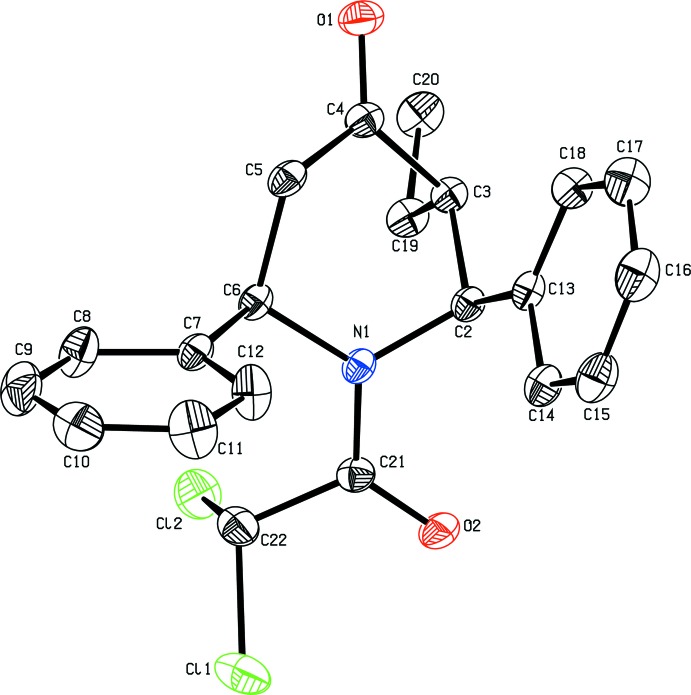
The molecular structure of the title compound, showing the atomic numbering and displacement ellipsoids drawn at 50% probability level.

**Fig. 2.**
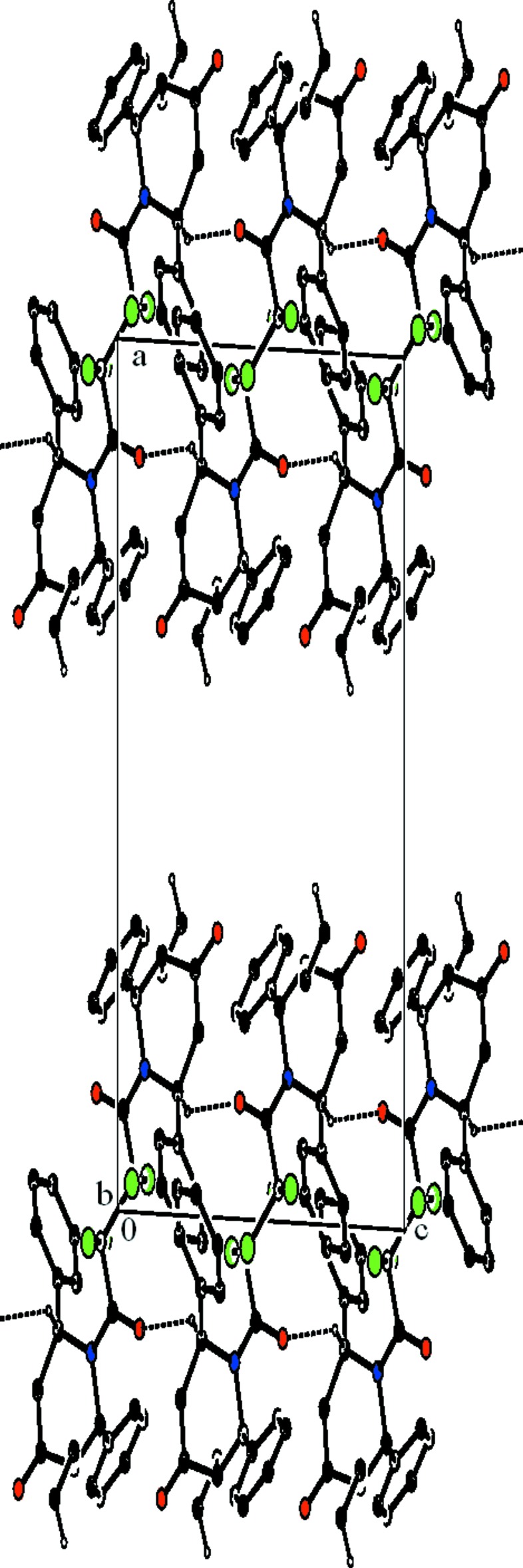
The crystal packing of the molecules. H atoms not involved in hydrogen bonding (dashed lines) have been omitted for clarity.

## supplementary crystallographic information

### Comment

Piperidine derivatives are the valued heterocyclic compounds in the field of medicinal chemistry. The compounds possessing an amide bond linkage have a wide range of biological activities such as antimicrobial, anti-inflammatory, antiviral, antimalarial and general anesthetics (Aridoss *et al.*, 2009). Functionalized piperidines are familiar substructures found in biologically active natural products and synthetic pharmaceuticals (Michael, 2001; Pinder, 1992; Rubiralta *et al.*, 1991). Piperidines have been found to exhibit blood cholesterol-lowering activities (Nalanishi *et al.*, 1974). Against this background and to ascertain the molecular structure and conformation, crystallographic study of the title compound has been carried out.

The *ORTEP* plot of the molecule is shown in Fig. 1. The chloro substituted piperidine derivative crystallizes in monoclinic space group *P*2_1_/*c*. The piperidine ring adopts distorted boat conformation with the puckering parameters (Cremer & Pople, 1975) and the asymmetry parameters (Nardelli, 1983) are: q_2_=0.651 (3) Å, q_3_ = -0.078 (3) Å, φ_2_ = 77.0 (2)° and Δ_s_(C2 & C5)= 19.1 (2)°. The sum of the bond angles around N1 [359°] is in accordance with *sp^2^* hybridization.

The planar phenyl rings [C7—C12 & C13—C18] substituted at 2,6-positions of the piperidine ring subtend angles of 65.4 (1)° [64.7 (2)°] & 89.2 (1)° [86.3 (2)°] in both the molecules with the best plane of the piperidine ring. The two phenyl rings are approximately perpendicular to each other with a dihedral angle of 86.5 (1)° [82.8 (2)°].

The dichloroacetyl groups substituted at the N^th^-position of the piperidine ring are *axial* and *equatorial* orientation for the both molecules which can be seen from the torsion angles of [N1—C21—C22—Cl2=] 88.5 (2)° [-97.0 (2)°] & [N1—C21—C22—Cl1=] -150.5 (2)° [142.5 (2)°], respectively.

The crystal packing reveals that the symmetry related molecules are linked through a network of C—H···O type of intermolecular interactions.

### Experimental

3-Ethyl-2,6-diphenylpiperidin-4-one (5 mmol) was dissolved in 60 ml of anhydrous benzene. To this solution, dichloroacetylchloride (20 mmol) and triethylamine (20 mmol) were added and the reaction mixture was allowed to stirr for 8 h. The course of the reaction was monitored by TLC. The organic layer was dried over anhydrous Na_2_SO_4_ and the resulting pasty mass was purified by recrystallization from ethyl acetate. Yield: 74%, Melting point: 140–142°C

### Refinement

N and C-bound H atoms were positioned geometrically (C–H = 0.93–0.98 Å) and allowed to ride on their parent atoms, with *U*_iso_(H) = 1.5*U*_eq_(C) for methyl H atoms and 1.2*U*_eq_(C) for all other H atoms.

### Crystal data

**Table:**

C_21_H_21_Cl_2_NO_2_	*F*(000) = 1632
*M**_r_* = 390.29	*D*_x_ = 1.306 Mg m^−^^3^
Monoclinic, *P*2_1_/*c*	Mo *K*α radiation, λ = 0.71073 Å
Hall symbol: -P 2ybc	Cell parameters from 5029 reflections
*a* = 17.4615 (13) Å	θ = 1.2–28.5°
*b* = 19.0291 (15) Å	µ = 0.34 mm^−^^1^
*c* = 11.9501 (9) Å	*T* = 293 K
β = 91.825 (5)°	Block, yellow
*V* = 3968.7 (5) Å^3^	0.22 × 0.20 × 0.18 mm
*Z* = 8	

### Data collection

**Table:**

Bruker SMART APEXII CCD diffractometer	9905 independent reflections
Radiation source: fine-focus sealed tube	5029 reflections with *I* > 2σ(*I*)
Graphite monochromator	*R*_int_ = 0.058
ω and φ scans	θ_max_ = 28.5°, θ_min_ = 1.2°
Absorption correction: multi-scan (*SADABS*; Bruker, 2008)	*h* = −23→22
*T*_min_ = 0.928, *T*_max_ = 0.940	*k* = −25→25
36640 measured reflections	*l* = −15→15

### Refinement

**Table:**

Refinement on *F*^2^	Primary atom site location: structure-invariant direct methods
Least-squares matrix: full	Secondary atom site location: difference Fourier map
*R*[*F*^2^ > 2σ(*F*^2^)] = 0.054	Hydrogen site location: inferred from neighbouring sites
*wR*(*F*^2^) = 0.175	H-atom parameters constrained
*S* = 1.00	*w* = 1/[σ^2^(*F*_o_^2^) + (0.0716*P*)^2^ + 1.3041*P*] where *P* = (*F*_o_^2^ + 2*F*_c_^2^)/3
9905 reflections	(Δ/σ)_max_ = 0.001
471 parameters	Δρ_max_ = 0.40 e Å^−^^3^
0 restraints	Δρ_min_ = −0.44 e Å^−^^3^

### Special details

**Table:**

Geometry. All e.s.d.'s (except the e.s.d. in the dihedral angle between two l.s. planes) are estimated using the full covariance matrix. The cell e.s.d.'s are taken into account individually in the estimation of e.s.d.'s in distances, angles and torsion angles; correlations between e.s.d.'s in cell parameters are only used when they are defined by crystal symmetry. An approximate (isotropic) treatment of cell e.s.d.'s is used for estimating e.s.d.'s involving l.s. planes.
Refinement. Refinement of *F*^2^ against ALL reflections. The weighted *R*-factor *wR* and goodness of fit *S* are based on *F*^2^, conventional *R*-factors *R* are based on *F*, with *F* set to zero for negative *F*^2^. The threshold expression of *F*^2^ > σ(*F*^2^) is used only for calculating *R*-factors(gt) *etc*. and is not relevant to the choice of reflections for refinement. *R*-factors based on *F*^2^ are statistically about twice as large as those based on *F*, and *R*- factors based on ALL data will be even larger.

### Fractional atomic coordinates and isotropic or equivalent isotropic displacement parameters (Å^2^)

**Table:**

	*x*	*y*	*z*	*U*_iso_*/*U*_eq_	
C2'	0.75368 (14)	0.67575 (13)	0.9369 (2)	0.0483 (6)	
H2'	0.7482	0.7032	1.0055	0.058*	
C2	0.56922 (13)	0.31595 (12)	0.33385 (18)	0.0395 (5)	
H2	0.5610	0.2915	0.2623	0.047*	
C3'	0.70448 (15)	0.71253 (14)	0.8467 (2)	0.0547 (7)	
H3'	0.6505	0.7058	0.8638	0.066*	
C3	0.53120 (14)	0.27084 (12)	0.42207 (19)	0.0426 (5)	
H3	0.4757	0.2722	0.4077	0.051*	
C4	0.54816 (14)	0.30054 (12)	0.5380 (2)	0.0445 (6)	
C4'	0.71760 (15)	0.68289 (14)	0.7315 (2)	0.0548 (7)	
C5	0.61719 (14)	0.34645 (13)	0.54913 (19)	0.0465 (6)	
H5A	0.6365	0.3437	0.6260	0.056*	
H5B	0.6011	0.3946	0.5357	0.056*	
C5'	0.78880 (14)	0.64026 (15)	0.7192 (2)	0.0522 (6)	
H5'1	0.8029	0.6420	0.6414	0.063*	
H5'2	0.7770	0.5917	0.7365	0.063*	
C6'	0.85851 (14)	0.66251 (13)	0.79195 (18)	0.0448 (6)	
H6'	0.8805	0.7044	0.7577	0.054*	
C6	0.68376 (13)	0.33050 (12)	0.47226 (18)	0.0407 (5)	
H6	0.7091	0.2875	0.4992	0.049*	
C7	0.74120 (14)	0.39014 (13)	0.48152 (19)	0.0445 (6)	
C7'	0.91814 (14)	0.60476 (14)	0.7896 (2)	0.0500 (6)	
C8	0.80352 (18)	0.38388 (17)	0.5544 (2)	0.0701 (8)	
H8	0.8120	0.3418	0.5924	0.084*	
C8'	0.97601 (17)	0.60923 (18)	0.7143 (3)	0.0732 (9)	
H8'	0.9792	0.6486	0.6687	0.088*	
C9'	1.0291 (2)	0.5564 (3)	0.7056 (4)	0.1019 (14)	
H9'	1.0679	0.5602	0.6543	0.122*	
C9	0.8536 (2)	0.4392 (2)	0.5716 (3)	0.0848 (10)	
H9	0.8952	0.4341	0.6216	0.102*	
C10'	1.0252 (2)	0.4985 (3)	0.7719 (4)	0.1046 (15)	
H10'	1.0611	0.4627	0.7657	0.126*	
C10	0.84274 (19)	0.50106 (19)	0.5162 (3)	0.0758 (9)	
H10	0.8761	0.5386	0.5289	0.091*	
C11	0.7828 (2)	0.50713 (17)	0.4426 (3)	0.0823 (10)	
H11	0.7758	0.5488	0.4029	0.099*	
C11'	0.9687 (3)	0.4928 (2)	0.8474 (3)	0.1001 (13)	
H11'	0.9662	0.4534	0.8932	0.120*	
C12	0.73198 (18)	0.45266 (15)	0.4252 (3)	0.0693 (8)	
H12	0.6908	0.4583	0.3747	0.083*	
C12'	0.9150 (2)	0.54601 (17)	0.8556 (3)	0.0777 (9)	
H12'	0.8762	0.5418	0.9067	0.093*	
C13'	0.73454 (14)	0.60042 (14)	0.9668 (2)	0.0508 (6)	
C13	0.53655 (14)	0.38928 (12)	0.31778 (19)	0.0432 (5)	
C14'	0.77351 (17)	0.56980 (16)	1.0579 (2)	0.0634 (7)	
H14'	0.8075	0.5970	1.1008	0.076*	
C14	0.57078 (15)	0.43437 (14)	0.2430 (2)	0.0542 (6)	
H14	0.6127	0.4189	0.2037	0.065*	
C15	0.54368 (19)	0.50191 (15)	0.2260 (2)	0.0667 (8)	
H15	0.5677	0.5317	0.1764	0.080*	
C15'	0.76317 (19)	0.50055 (18)	1.0861 (3)	0.0743 (9)	
H15'	0.7903	0.4814	1.1471	0.089*	
C16	0.48117 (19)	0.52479 (16)	0.2825 (3)	0.0698 (8)	
H16	0.4634	0.5705	0.2725	0.084*	
C16'	0.7127 (2)	0.45959 (18)	1.0242 (3)	0.0764 (9)	
H16'	0.7064	0.4123	1.0413	0.092*	
C17	0.44518 (18)	0.48050 (15)	0.3530 (3)	0.0685 (8)	
H17	0.4017	0.4957	0.3889	0.082*	
C17'	0.6718 (2)	0.48940 (19)	0.9369 (3)	0.0831 (10)	
H17'	0.6366	0.4623	0.8958	0.100*	
C18	0.47247 (15)	0.41297 (14)	0.3722 (2)	0.0539 (6)	
H18	0.4478	0.3835	0.4217	0.065*	
C18'	0.68197 (17)	0.55937 (17)	0.9089 (3)	0.0673 (8)	
H18'	0.6529	0.5788	0.8501	0.081*	
C19'	0.72176 (19)	0.79250 (16)	0.8463 (3)	0.0743 (9)	
H19A	0.7229	0.8093	0.9230	0.089*	
H19B	0.7724	0.7997	0.8171	0.089*	
C19	0.55729 (16)	0.19375 (13)	0.4187 (2)	0.0510 (6)	
H19C	0.5588	0.1785	0.3414	0.061*	
H19D	0.6088	0.1902	0.4511	0.061*	
C20	0.50454 (18)	0.14531 (14)	0.4820 (2)	0.0640 (7)	
H20A	0.5051	0.1585	0.5595	0.096*	
H20B	0.5219	0.0977	0.4754	0.096*	
H20C	0.4533	0.1491	0.4509	0.096*	
C20'	0.6652 (2)	0.83623 (19)	0.7787 (3)	0.0905 (11)	
H20D	0.6618	0.8189	0.7033	0.136*	
H20E	0.6820	0.8843	0.7783	0.136*	
H20F	0.6158	0.8334	0.8114	0.136*	
C21'	0.88364 (15)	0.71449 (14)	0.9815 (2)	0.0545 (7)	
C21	0.69852 (14)	0.29727 (13)	0.2715 (2)	0.0438 (5)	
C22	0.78180 (14)	0.27937 (15)	0.3034 (2)	0.0562 (7)	
H22	0.7985	0.3075	0.3685	0.067*	
C22'	0.96346 (16)	0.73251 (16)	0.9400 (2)	0.0627 (8)	
H22'	0.9753	0.7022	0.8765	0.075*	
N1'	0.83558 (11)	0.68196 (10)	0.90685 (16)	0.0430 (5)	
N1	0.65366 (11)	0.31767 (9)	0.35573 (15)	0.0389 (4)	
O1'	0.67356 (12)	0.69236 (13)	0.65272 (18)	0.0795 (6)	
O1	0.50727 (11)	0.28989 (10)	0.61640 (15)	0.0603 (5)	
O2'	0.86636 (12)	0.73129 (13)	1.07511 (17)	0.0803 (7)	
O2	0.67618 (10)	0.29028 (10)	0.17464 (14)	0.0577 (5)	
Cl1'	0.96162 (6)	0.82083 (5)	0.89659 (10)	0.1063 (4)	
Cl1	0.84132 (5)	0.29718 (5)	0.19055 (8)	0.0855 (3)	
Cl2	0.78382 (5)	0.18921 (4)	0.33926 (7)	0.0781 (3)	
Cl2'	1.03240 (5)	0.72012 (6)	1.04814 (9)	0.0994 (3)	

### Atomic displacement parameters (Å^2^)

**Table:**

	*U*^11^	*U*^22^	*U*^33^	*U*^12^	*U*^13^	*U*^23^
C2'	0.0476 (14)	0.0536 (16)	0.0442 (13)	0.0010 (11)	0.0076 (10)	−0.0025 (11)
C2	0.0465 (13)	0.0371 (13)	0.0346 (12)	0.0006 (10)	−0.0015 (9)	−0.0047 (9)
C3'	0.0462 (15)	0.0575 (17)	0.0607 (16)	0.0018 (12)	0.0056 (11)	0.0051 (13)
C3	0.0484 (14)	0.0377 (13)	0.0417 (13)	0.0022 (10)	0.0023 (10)	−0.0017 (10)
C4	0.0542 (15)	0.0382 (13)	0.0413 (13)	0.0127 (11)	0.0064 (10)	0.0001 (10)
C4'	0.0475 (15)	0.0567 (17)	0.0596 (17)	−0.0098 (12)	−0.0053 (12)	0.0081 (13)
C5	0.0628 (16)	0.0429 (14)	0.0339 (12)	0.0063 (12)	0.0014 (10)	−0.0066 (10)
C5'	0.0570 (16)	0.0575 (17)	0.0418 (13)	−0.0030 (13)	−0.0023 (11)	−0.0031 (12)
C6'	0.0501 (14)	0.0471 (14)	0.0373 (12)	−0.0030 (11)	0.0040 (10)	−0.0006 (10)
C6	0.0519 (14)	0.0365 (13)	0.0332 (11)	0.0030 (10)	−0.0033 (9)	−0.0020 (9)
C7	0.0543 (15)	0.0436 (14)	0.0354 (12)	−0.0001 (11)	−0.0020 (10)	−0.0084 (10)
C7'	0.0510 (15)	0.0590 (17)	0.0399 (13)	0.0004 (12)	0.0005 (10)	−0.0086 (12)
C8	0.083 (2)	0.070 (2)	0.0563 (17)	−0.0106 (17)	−0.0196 (15)	0.0026 (15)
C8'	0.0600 (18)	0.082 (2)	0.078 (2)	−0.0036 (16)	0.0163 (15)	−0.0175 (17)
C9'	0.065 (2)	0.121 (4)	0.122 (3)	0.008 (2)	0.020 (2)	−0.052 (3)
C9	0.082 (2)	0.102 (3)	0.069 (2)	−0.023 (2)	−0.0250 (17)	−0.013 (2)
C10'	0.080 (3)	0.107 (3)	0.124 (4)	0.041 (2)	−0.029 (2)	−0.057 (3)
C10	0.075 (2)	0.065 (2)	0.087 (2)	−0.0167 (17)	0.0042 (18)	−0.0323 (19)
C11	0.085 (2)	0.0466 (18)	0.114 (3)	−0.0094 (17)	−0.013 (2)	0.0011 (18)
C11'	0.129 (3)	0.083 (3)	0.088 (3)	0.046 (3)	−0.010 (2)	−0.007 (2)
C12	0.070 (2)	0.0474 (17)	0.089 (2)	−0.0043 (14)	−0.0236 (16)	0.0057 (15)
C12'	0.098 (2)	0.073 (2)	0.0621 (19)	0.0297 (19)	0.0124 (16)	0.0063 (16)
C13'	0.0490 (15)	0.0573 (16)	0.0468 (14)	−0.0031 (12)	0.0101 (11)	0.0041 (12)
C13	0.0514 (14)	0.0401 (13)	0.0374 (12)	0.0008 (11)	−0.0079 (10)	−0.0011 (10)
C14'	0.0730 (19)	0.068 (2)	0.0495 (16)	−0.0082 (15)	0.0004 (13)	0.0079 (14)
C14	0.0627 (17)	0.0513 (16)	0.0481 (14)	0.0032 (13)	−0.0053 (12)	0.0071 (12)
C15	0.088 (2)	0.0491 (17)	0.0618 (18)	−0.0020 (16)	−0.0123 (16)	0.0153 (14)
C15'	0.084 (2)	0.079 (2)	0.0598 (18)	−0.0006 (18)	0.0047 (16)	0.0254 (17)
C16	0.094 (2)	0.0417 (16)	0.072 (2)	0.0169 (16)	−0.0134 (17)	0.0017 (15)
C16'	0.087 (2)	0.066 (2)	0.077 (2)	−0.0142 (18)	0.0154 (18)	0.0180 (17)
C17	0.080 (2)	0.0495 (17)	0.076 (2)	0.0179 (15)	−0.0008 (16)	−0.0094 (15)
C17'	0.090 (2)	0.076 (2)	0.083 (2)	−0.0334 (19)	−0.0022 (19)	0.0125 (19)
C18	0.0626 (17)	0.0456 (15)	0.0536 (15)	0.0053 (13)	0.0018 (12)	−0.0013 (12)
C18'	0.0635 (18)	0.072 (2)	0.0665 (18)	−0.0177 (16)	−0.0031 (14)	0.0155 (16)
C19'	0.078 (2)	0.0592 (19)	0.085 (2)	0.0062 (16)	−0.0018 (17)	0.0047 (17)
C19	0.0650 (17)	0.0385 (14)	0.0496 (15)	0.0041 (12)	0.0052 (12)	−0.0018 (11)
C20	0.083 (2)	0.0454 (16)	0.0637 (18)	−0.0069 (14)	0.0018 (14)	0.0047 (14)
C20'	0.077 (2)	0.076 (2)	0.119 (3)	0.0138 (18)	0.008 (2)	0.030 (2)
C21'	0.0580 (16)	0.0532 (16)	0.0520 (15)	0.0002 (13)	−0.0031 (12)	−0.0140 (13)
C21	0.0490 (14)	0.0409 (13)	0.0417 (13)	−0.0037 (11)	0.0041 (10)	−0.0083 (10)
C22	0.0491 (15)	0.0607 (18)	0.0590 (16)	−0.0012 (13)	0.0048 (12)	−0.0228 (13)
C22'	0.0569 (17)	0.0647 (19)	0.0661 (18)	−0.0093 (14)	−0.0053 (13)	−0.0183 (14)
N1'	0.0466 (11)	0.0438 (12)	0.0388 (10)	−0.0011 (9)	0.0040 (8)	−0.0037 (9)
N1	0.0469 (11)	0.0368 (11)	0.0329 (10)	0.0015 (8)	−0.0019 (8)	−0.0050 (8)
O1'	0.0639 (13)	0.1041 (18)	0.0690 (14)	0.0004 (12)	−0.0230 (10)	0.0063 (12)
O1	0.0666 (12)	0.0679 (13)	0.0472 (10)	0.0088 (9)	0.0160 (9)	0.0014 (9)
O2'	0.0758 (14)	0.1051 (18)	0.0600 (13)	−0.0062 (12)	0.0022 (10)	−0.0376 (12)
O2	0.0613 (11)	0.0755 (13)	0.0363 (10)	−0.0051 (9)	0.0038 (8)	−0.0140 (9)
Cl1'	0.1115 (8)	0.0779 (6)	0.1289 (9)	−0.0250 (5)	−0.0043 (6)	0.0173 (6)
Cl1	0.0628 (5)	0.0953 (6)	0.1002 (6)	−0.0129 (4)	0.0303 (4)	−0.0139 (5)
Cl2	0.0760 (5)	0.0715 (5)	0.0868 (6)	0.0192 (4)	0.0013 (4)	0.0032 (4)
Cl2'	0.0690 (6)	0.1191 (8)	0.1083 (7)	−0.0001 (5)	−0.0281 (5)	−0.0051 (6)

### Geometric parameters (Å, º)

**Table:**

C2'—N1'	1.490 (3)	C11'—H11'	0.9300
C2'—C13'	1.517 (4)	C12—H12	0.9300
C2'—C3'	1.527 (4)	C12'—H12'	0.9300
C2'—H2'	0.9800	C13'—C18'	1.375 (4)
C2—N1	1.489 (3)	C13'—C14'	1.393 (4)
C2—C13	1.517 (3)	C13—C18	1.387 (3)
C2—C3	1.528 (3)	C13—C14	1.388 (3)
C2—H2	0.9800	C14'—C15'	1.374 (4)
C3'—C4'	1.512 (4)	C14'—H14'	0.9300
C3'—C19'	1.551 (4)	C14—C15	1.382 (4)
C3'—H3'	0.9800	C14—H14	0.9300
C3—C4	1.516 (3)	C15—C16	1.372 (4)
C3—C19	1.537 (3)	C15—H15	0.9300
C3—H3	0.9800	C15'—C16'	1.376 (5)
C4—O1	1.213 (3)	C15'—H15'	0.9300
C4—C5	1.491 (4)	C16—C17	1.360 (4)
C4'—O1'	1.210 (3)	C16—H16	0.9300
C4'—C5'	1.496 (4)	C16'—C17'	1.368 (5)
C5—C6	1.535 (3)	C16'—H16'	0.9300
C5—H5A	0.9700	C17—C18	1.387 (4)
C5—H5B	0.9700	C17—H17	0.9300
C5'—C6'	1.533 (3)	C17'—C18'	1.386 (4)
C5'—H5'1	0.9700	C17'—H17'	0.9300
C5'—H5'2	0.9700	C18—H18	0.9300
C6'—N1'	1.489 (3)	C18'—H18'	0.9300
C6'—C7'	1.515 (4)	C19'—C20'	1.506 (4)
C6'—H6'	0.9800	C19'—H19A	0.9700
C6—N1	1.493 (3)	C19'—H19B	0.9700
C6—C7	1.516 (3)	C19—C20	1.521 (4)
C6—H6	0.9800	C19—H19C	0.9700
C7—C12	1.374 (4)	C19—H19D	0.9700
C7—C8	1.377 (4)	C20—H20A	0.9600
C7'—C12'	1.370 (4)	C20—H20B	0.9600
C7'—C8'	1.377 (4)	C20—H20C	0.9600
C8—C9	1.379 (4)	C20'—H20D	0.9600
C8—H8	0.9300	C20'—H20E	0.9600
C8'—C9'	1.375 (5)	C20'—H20F	0.9600
C8'—H8'	0.9300	C21'—O2'	1.210 (3)
C9'—C10'	1.360 (6)	C21'—N1'	1.355 (3)
C9'—H9'	0.9300	C21'—C22'	1.533 (4)
C9—C10	1.362 (5)	C21—O2	1.217 (3)
C9—H9	0.9300	C21—N1	1.352 (3)
C10'—C11'	1.362 (6)	C21—C22	1.530 (4)
C10'—H10'	0.9300	C22—Cl1	1.762 (3)
C10—C11	1.350 (5)	C22—Cl2	1.768 (3)
C10—H10	0.9300	C22—H22	0.9800
C11—C12	1.376 (4)	C22'—Cl2'	1.753 (3)
C11—H11	0.9300	C22'—Cl1'	1.759 (3)
C11'—C12'	1.387 (5)	C22'—H22'	0.9800
			
N1'—C2'—C13'	110.6 (2)	C7'—C12'—C11'	121.0 (3)
N1'—C2'—C3'	108.43 (19)	C7'—C12'—H12'	119.5
C13'—C2'—C3'	118.4 (2)	C11'—C12'—H12'	119.5
N1'—C2'—H2'	106.2	C18'—C13'—C14'	117.2 (3)
C13'—C2'—H2'	106.2	C18'—C13'—C2'	124.5 (2)
C3'—C2'—H2'	106.2	C14'—C13'—C2'	118.3 (2)
N1—C2—C13	111.60 (18)	C18—C13—C14	118.1 (2)
N1—C2—C3	109.92 (18)	C18—C13—C2	123.0 (2)
C13—C2—C3	115.73 (19)	C14—C13—C2	118.8 (2)
N1—C2—H2	106.3	C15'—C14'—C13'	121.9 (3)
C13—C2—H2	106.3	C15'—C14'—H14'	119.0
C3—C2—H2	106.3	C13'—C14'—H14'	119.0
C4'—C3'—C2'	112.0 (2)	C15—C14—C13	121.1 (3)
C4'—C3'—C19'	109.2 (2)	C15—C14—H14	119.4
C2'—C3'—C19'	110.3 (2)	C13—C14—H14	119.4
C4'—C3'—H3'	108.5	C16—C15—C14	119.8 (3)
C2'—C3'—H3'	108.5	C16—C15—H15	120.1
C19'—C3'—H3'	108.5	C14—C15—H15	120.1
C4—C3—C2	110.14 (19)	C14'—C15'—C16'	119.9 (3)
C4—C3—C19	109.3 (2)	C14'—C15'—H15'	120.1
C2—C3—C19	112.50 (19)	C16'—C15'—H15'	120.1
C4—C3—H3	108.3	C17—C16—C15	119.9 (3)
C2—C3—H3	108.3	C17—C16—H16	120.0
C19—C3—H3	108.3	C15—C16—H16	120.0
O1—C4—C5	121.5 (2)	C17'—C16'—C15'	119.0 (3)
O1—C4—C3	122.7 (2)	C17'—C16'—H16'	120.5
C5—C4—C3	115.7 (2)	C15'—C16'—H16'	120.5
O1'—C4'—C5'	121.0 (3)	C16—C17—C18	120.9 (3)
O1'—C4'—C3'	122.8 (3)	C16—C17—H17	119.6
C5'—C4'—C3'	116.2 (2)	C18—C17—H17	119.6
C4—C5—C6	117.13 (19)	C16'—C17'—C18'	121.0 (3)
C4—C5—H5A	108.0	C16'—C17'—H17'	119.5
C6—C5—H5A	108.0	C18'—C17'—H17'	119.5
C4—C5—H5B	108.0	C17—C18—C13	120.1 (3)
C6—C5—H5B	108.0	C17—C18—H18	120.0
H5A—C5—H5B	107.3	C13—C18—H18	120.0
C4'—C5'—C6'	116.3 (2)	C13'—C18'—C17'	120.8 (3)
C4'—C5'—H5'1	108.2	C13'—C18'—H18'	119.6
C6'—C5'—H5'1	108.2	C17'—C18'—H18'	119.6
C4'—C5'—H5'2	108.2	C20'—C19'—C3'	114.8 (3)
C6'—C5'—H5'2	108.2	C20'—C19'—H19A	108.6
H5'1—C5'—H5'2	107.4	C3'—C19'—H19A	108.6
N1'—C6'—C7'	113.72 (19)	C20'—C19'—H19B	108.6
N1'—C6'—C5'	111.07 (19)	C3'—C19'—H19B	108.6
C7'—C6'—C5'	108.9 (2)	H19A—C19'—H19B	107.5
N1'—C6'—H6'	107.7	C20—C19—C3	112.4 (2)
C7'—C6'—H6'	107.7	C20—C19—H19C	109.1
C5'—C6'—H6'	107.7	C3—C19—H19C	109.1
N1—C6—C7	113.76 (18)	C20—C19—H19D	109.1
N1—C6—C5	109.86 (19)	C3—C19—H19D	109.1
C7—C6—C5	108.67 (18)	H19C—C19—H19D	107.9
N1—C6—H6	108.1	C19—C20—H20A	109.5
C7—C6—H6	108.1	C19—C20—H20B	109.5
C5—C6—H6	108.1	H20A—C20—H20B	109.5
C12—C7—C8	117.5 (3)	C19—C20—H20C	109.5
C12—C7—C6	123.1 (2)	H20A—C20—H20C	109.5
C8—C7—C6	119.3 (2)	H20B—C20—H20C	109.5
C12'—C7'—C8'	118.1 (3)	C19'—C20'—H20D	109.5
C12'—C7'—C6'	122.8 (2)	C19'—C20'—H20E	109.5
C8'—C7'—C6'	119.1 (3)	H20D—C20'—H20E	109.5
C7—C8—C9	120.9 (3)	C19'—C20'—H20F	109.5
C7—C8—H8	119.6	H20D—C20'—H20F	109.5
C9—C8—H8	119.6	H20E—C20'—H20F	109.5
C9'—C8'—C7'	121.0 (4)	O2'—C21'—N1'	124.2 (3)
C9'—C8'—H8'	119.5	O2'—C21'—C22'	119.7 (2)
C7'—C8'—H8'	119.5	N1'—C21'—C22'	116.1 (2)
C10'—C9'—C8'	120.1 (4)	O2—C21—N1	124.3 (2)
C10'—C9'—H9'	119.9	O2—C21—C22	119.1 (2)
C8'—C9'—H9'	119.9	N1—C21—C22	116.5 (2)
C10—C9—C8	120.6 (3)	C21—C22—Cl1	110.20 (19)
C10—C9—H9	119.7	C21—C22—Cl2	106.73 (17)
C8—C9—H9	119.7	Cl1—C22—Cl2	111.37 (14)
C9'—C10'—C11'	120.1 (4)	C21—C22—H22	109.5
C9'—C10'—H10'	120.0	Cl1—C22—H22	109.5
C11'—C10'—H10'	120.0	Cl2—C22—H22	109.5
C11—C10—C9	118.9 (3)	C21'—C22'—Cl2'	110.0 (2)
C11—C10—H10	120.5	C21'—C22'—Cl1'	107.5 (2)
C9—C10—H10	120.5	Cl2'—C22'—Cl1'	110.59 (15)
C10—C11—C12	121.1 (3)	C21'—C22'—H22'	109.6
C10—C11—H11	119.5	Cl2'—C22'—H22'	109.6
C12—C11—H11	119.5	Cl1'—C22'—H22'	109.6
C10'—C11'—C12'	119.7 (4)	C21'—N1'—C6'	122.8 (2)
C10'—C11'—H11'	120.2	C21'—N1'—C2'	117.16 (19)
C12'—C11'—H11'	120.2	C6'—N1'—C2'	119.39 (19)
C7—C12—C11	120.9 (3)	C21—N1—C2	117.17 (18)
C7—C12—H12	119.5	C21—N1—C6	123.13 (19)
C11—C12—H12	119.5	C2—N1—C6	118.98 (17)
			
N1'—C2'—C3'—C4'	56.8 (3)	N1—C2—C13—C14	50.1 (3)
C13'—C2'—C3'—C4'	−70.3 (3)	C3—C2—C13—C14	176.8 (2)
N1'—C2'—C3'—C19'	−65.0 (3)	C18'—C13'—C14'—C15'	3.1 (4)
C13'—C2'—C3'—C19'	168.0 (2)	C2'—C13'—C14'—C15'	−175.8 (3)
N1—C2—C3—C4	59.4 (2)	C18—C13—C14—C15	2.3 (4)
C13—C2—C3—C4	−68.1 (3)	C2—C13—C14—C15	−179.5 (2)
N1—C2—C3—C19	−62.8 (2)	C13—C14—C15—C16	−1.0 (4)
C13—C2—C3—C19	169.7 (2)	C13'—C14'—C15'—C16'	−0.5 (5)
C2—C3—C4—O1	155.8 (2)	C14—C15—C16—C17	−1.4 (5)
C19—C3—C4—O1	−80.2 (3)	C14'—C15'—C16'—C17'	−1.9 (5)
C2—C3—C4—C5	−21.9 (3)	C15—C16—C17—C18	2.5 (5)
C19—C3—C4—C5	102.1 (2)	C15'—C16'—C17'—C18'	1.5 (5)
C2'—C3'—C4'—O1'	162.7 (3)	C16—C17—C18—C13	−1.1 (4)
C19'—C3'—C4'—O1'	−74.9 (3)	C14—C13—C18—C17	−1.3 (4)
C2'—C3'—C4'—C5'	−17.0 (3)	C2—C13—C18—C17	−179.5 (2)
C19'—C3'—C4'—C5'	105.4 (3)	C14'—C13'—C18'—C17'	−3.5 (4)
O1—C4—C5—C6	151.5 (2)	C2'—C13'—C18'—C17'	175.4 (3)
C3—C4—C5—C6	−30.8 (3)	C16'—C17'—C18'—C13'	1.2 (5)
O1'—C4'—C5'—C6'	147.2 (3)	C4'—C3'—C19'—C20'	69.2 (3)
C3'—C4'—C5'—C6'	−33.1 (3)	C2'—C3'—C19'—C20'	−167.4 (3)
C4'—C5'—C6'—N1'	40.9 (3)	C4—C3—C19—C20	73.3 (3)
C4'—C5'—C6'—C7'	166.8 (2)	C2—C3—C19—C20	−164.0 (2)
C4—C5—C6—N1	44.8 (3)	O2—C21—C22—Cl1	31.9 (3)
C4—C5—C6—C7	169.9 (2)	N1—C21—C22—Cl1	−150.47 (18)
N1—C6—C7—C12	42.3 (3)	O2—C21—C22—Cl2	−89.1 (3)
C5—C6—C7—C12	−80.4 (3)	N1—C21—C22—Cl2	88.5 (2)
N1—C6—C7—C8	−141.7 (2)	O2'—C21'—C22'—Cl2'	39.6 (3)
C5—C6—C7—C8	95.6 (3)	N1'—C21'—C22'—Cl2'	−142.5 (2)
N1'—C6'—C7'—C12'	43.1 (4)	O2'—C21'—C22'—Cl1'	−80.9 (3)
C5'—C6'—C7'—C12'	−81.3 (3)	N1'—C21'—C22'—Cl1'	97.0 (2)
N1'—C6'—C7'—C8'	−140.4 (2)	O2'—C21'—N1'—C6'	179.5 (3)
C5'—C6'—C7'—C8'	95.2 (3)	C22'—C21'—N1'—C6'	1.7 (4)
C12—C7—C8—C9	1.4 (4)	O2'—C21'—N1'—C2'	8.8 (4)
C6—C7—C8—C9	−174.8 (3)	C22'—C21'—N1'—C2'	−169.0 (2)
C12'—C7'—C8'—C9'	−0.1 (5)	C7'—C6'—N1'—C21'	68.2 (3)
C6'—C7'—C8'—C9'	−176.8 (3)	C5'—C6'—N1'—C21'	−168.6 (2)
C7'—C8'—C9'—C10'	0.0 (6)	C7'—C6'—N1'—C2'	−121.3 (2)
C7—C8—C9—C10	−0.5 (5)	C5'—C6'—N1'—C2'	1.9 (3)
C8'—C9'—C10'—C11'	−0.2 (6)	C13'—C2'—N1'—C21'	−107.6 (2)
C8—C9—C10—C11	−1.1 (5)	C3'—C2'—N1'—C21'	121.0 (2)
C9—C10—C11—C12	1.7 (6)	C13'—C2'—N1'—C6'	81.4 (3)
C9'—C10'—C11'—C12'	0.5 (6)	C3'—C2'—N1'—C6'	−50.1 (3)
C8—C7—C12—C11	−0.9 (5)	O2—C21—N1—C2	14.1 (3)
C6—C7—C12—C11	175.2 (3)	C22—C21—N1—C2	−163.3 (2)
C10—C11—C12—C7	−0.7 (5)	O2—C21—N1—C6	−175.7 (2)
C8'—C7'—C12'—C11'	0.4 (5)	C22—C21—N1—C6	6.8 (3)
C6'—C7'—C12'—C11'	177.0 (3)	C13—C2—N1—C21	−105.9 (2)
C10'—C11'—C12'—C7'	−0.6 (6)	C3—C2—N1—C21	124.3 (2)
N1'—C2'—C13'—C18'	−117.4 (3)	C13—C2—N1—C6	83.6 (2)
C3'—C2'—C13'—C18'	8.6 (4)	C3—C2—N1—C6	−46.2 (2)
N1'—C2'—C13'—C14'	61.4 (3)	C7—C6—N1—C21	63.0 (3)
C3'—C2'—C13'—C14'	−172.6 (2)	C5—C6—N1—C21	−175.0 (2)
N1—C2—C13—C18	−131.7 (2)	C7—C6—N1—C2	−127.1 (2)
C3—C2—C13—C18	−5.0 (3)	C5—C6—N1—C2	−5.0 (3)

### Hydrogen-bond geometry (Å, º)

**Table:**

*D*—H···*A*	*D*—H	H···*A*	*D*···*A*	*D*—H···*A*
C2—H2···O1^i^	0.98	2.49	3.435 (3)	161
C20′—H20*F*···O1^ii^	0.96	2.48	3.415 (4)	164
C6′—H6′···O2′^iii^	0.98	2.51	3.292 (3)	137

Symmetry codes: (i) *x*, −*y*+1/2, *z*−1/2; (ii) −*x*+1, *y*+1/2, −*z*+3/2; (iii) *x*, −*y*+3/2, *z*−1/2.
